# Factors Predicting Completion of Four or More Antenatal Care Visits in Sarlahi District, Nepal

**DOI:** 10.21203/rs.3.rs-4467441/v1

**Published:** 2024-05-29

**Authors:** Yiwei Yue, Elizabeth A. Hazel, Seema Subedi, Scott Zeger, Diwakar Mohan, Luke C Mullany, James M Tielsch, Subarna K Khatry, Steven C. LeClerq, Joanne Katz

**Affiliations:** Department of International Health, Johns Hopkins Bloomberg School of Public Health; Department of International Health, Johns Hopkins Bloomberg School of Public Health; Department of International Health, Johns Hopkins Bloomberg School of Public Health; Department of Biostatistics, Johns Hopkins Bloomberg School of Public Health; Department of International Health, Johns Hopkins Bloomberg School of Public Health; Department of International Health, Johns Hopkins Bloomberg School of Public Health; Department of Global Health, Milken Institute School of Public Health, George Washington University, 950 New Hampshire Avenue; Nepal Nutrition Intervention Project, Sarlahi, 120 Sitakwa Margh, Jhamsikhel Pulchowk; Department of International Health, Johns Hopkins Bloomberg School of Public Health; Department of International Health, Johns Hopkins Bloomberg School of Public Health

**Keywords:** Maternal health, maternal and newborn healthcare, pregnancy, antenatal care, pregnancy care, obstetrics, public health, global health, risk factors, epidemiology, primary care, maternal mortality, pregnancy-related mortality, cluster randomized trial

## Abstract

**Background:**

A significant number of women die from pregnancy and childbirth complications globally, particularly in low- and middle-income countries (LMICs). Receiving at least four antenatal care (ANC) visits may be important in reducing maternal and perinatal deaths. This study investigates factors associated with attending ≥ 4 ANC visits in Sarlahi district of southern Nepal.

**Methods:**

A secondary analysis was conducted on data from the Nepal Oil Massage Study (NOMS), a cluster-randomized, community-based longitudinal pregnancy cohort study encompassing 34 Village Development Committees. We quantified the association between receipt/attendance of ≥ 4 ANC visits and socioeconomic, demographic, morbidity, and pregnancy history factors using logistic regression; Generalized Estimating Equations were used to account for multiple pregnancies per woman.

**Results:**

All pregnancies resulting in a live birth (n=31,867) were included in the model and 31.4% of those pregnancies received 4+ ANC visits. Significant positive associations include socioeconomic factors such as participation in non-farming occupations for women (OR=1.52, 95% CI: 1.19, 1.93), higher education (OR=1.79, 95% CI: 1.66, 1.93) and wealth quintile OR=1.44, 95% CI: 1.31, 1.59), nutritional status such as non-short stature (OR=1.17, 95% CI: 1.07, 1.27), obstetric history such as adequate interpregnancy interval (OR=1.31, 95% CI: 1.19, 1.45) and prior pregnancy but no live birth (OR=2.14, 95% CI: 1.57, 2.92), symptoms such as vaginal bleeding (OR=1.35, 95% CI:1.11, 1.65) and awareness of the government’s conditional cash transfer ANC program (OR=2.26, 95% CI: 2.01, 2.54). Conversely, belonging to the lower Shudra caste (OR=0.56, 95% CI: 0.47, 0.67), maternal age below 18 or above 35 (OR=0.81, 95% CI:0.74, 0.88; OR=0.77, 95% CI: 0.62, 0.96)), preterm birth (OR=0.41, 95% CI: 0.35, 0.49), parity ≥ 1 (OR=0.66, 95% CI: 0.61, 0.72), and the presence of hypertension during pregnancy (OR=0.79, 95% CI: 0.69, 0.90) were associated with decreased likelihood of attending ≥ 4 ANC visits.

**Conclusions:**

These findings underscore the importance of continuing and promoting the government’s program and increasing awareness among women. Moreover, understanding these factors can guide interventions aimed at encouraging ANC uptake in the most vulnerable groups, subsequently reducing maternal-related adverse outcomes in LMICs.

**Trial registration:**

The clinicaltrial.gov trial registration number for NOMS was #NCT01177111. Registration date was August 6^th^, 2010.

## Background

Despite improvements in global maternal health, the number of women dying due to pregnancy and childbirth complications in 2020 remains high, with estimates of 800 maternal deaths daily (1). Only 69% of pregnant women completed at least four antenatal care (ANC) visits globally in 2022 (1). Furthermore, around 95% of preventable maternal deaths occur in low- and middle-income countries (LMICs) in 2020, highlighting the disparities and inequalities in health services (1).

The 2016 Nepal Demographic and Health Survey (NDHS) reported a high pregnancy-related mortality ratio (PRMR) (259 maternal deaths per 100,000 live births) and a high maternal mortality ratio (MMR) (239 maternal deaths per 100,000 live births) during the 7 years preceding the survey (2). The levels of maternal mortality in Nepal have improved since 2016. Based on the Nepal Maternal Mortality Study 2021, the latest MMR was 151 maternal deaths per 100,000 live births in Nepal (3).

In addition, infant and child health improvement remained slow in Nepal between 2016 to 2022 (2, 4). The 2022 NDHS indicated that the neonatal mortality rate (NMR) remained the same as that of the 2016 NDHS (i.e. NMR: 21 deaths per 1,000 live births) (2, 4). The infant mortality rate (IMR) fell from 32 to 28 deaths per 1,000 live births) and the under-5 mortality rate (from 39 to 33 deaths per 1,000 live births) declined between 2016 and 2022 (2, 4).

According to the 2016 NDHS, ANC attendance and maternal health service utilization in Nepal were below the global average, with 84% of women attending any ANC visit with skilled providers, 69% with 4 + ANC visits, 57% delivered in a health facility, 58% delivery assisted by a skilled provider, and 57% postnatal care coverage (mothers and newborns received a postnatal check-up within 2 days of delivery by a skilled provided) in the 5 years before the survey (2). The 2022 NDHS showed improvements in maternal and newborn healthcare since 2016 (2, 4). In the two years prior to the 2022 survey, 94% of women had ANC from a skilled provider for their most recent live birth or stillbirth (4). Additionally, 80% of women attended ≥ 4 ANC visits, 79% of deliveries occurred in a health facility, 80% of deliveries were conducted by skilled providers, and 70% of women and newborns received a postnatal check (4).

In Nepal, there is an estimated 33% lower neonatal mortality among babies born to women attending at least four ANC sessions (5). Regular and quality ANC during pregnancy are important to detect and intervene in pregnancy risks, improve the health outcomes of pregnant women and newborns, and reduce maternal and neonatal mortality and morbidity (6–9). An adequate number of ANC visits and quality ANC services mitigate risks including anemia, pregnancy induced hypertension, preterm labor, preterm births and low birth weight (8, 10–12). ANC visits serve not only as a preventative measure but also provide education to women regarding childbirth, family planning, and birth spacing (13). Consequently, ANC encourages institutional deliveries, reduces maternal mortality, and fosters improved neonatal outcomes (13).

We aim to assess the association between demographic factors, socioeconomic status (SES), pregnancy history, morbidity symptoms in pregnancy, and the frequency of ANC visits by women in Sarlahi district of Nepal. These findings will inform the development of interventions to increase ANC visits and improve maternal and neonatal health in Nepal and possibly similar LMICs.

## Methods

### Study design

The Nepal Oil Massage Study (NOMS), was a cluster-randomized community-based controlled trial that included mothers and their live and stillborn infants in 34 Village Development Committees (VDCs) in Sarlahi District, rural southern Nepal, from 2010 to 2017 (14). A total of 340 geographic clusters were randomized to receive either sunflower seed oil to massage for newborn babies or the standard mustard seed oil (14). Married women of childbearing age were visited for pregnancy surveillance visits every 5 weeks and enrolled – with consent – when pregnant. Additional details about the NOMS study are available elsewhere (14).

### Participants

Since the trial enrolled and followed women over several years, some women contributed more than one pregnancy to the analysis. We excluded pregnancies resulting in fetal loss or records with missing values or stillbirths for pregnancy outcomes, only considering live births for analysis. All pregnancies that resulted in at least one live birth were included, regardless of their intervention assignment.

### Measures

The number of ANC visits women attended during pregnancy was obtained from maternal recall by study staff during home visits as soon as possible after delivery. Our primary ANC visit outcome was binarized as four or more ANC visits, and less than 4 ANC visits (including no visits).

Predictors including demographic, SES, and obstetric and medical history collected at enrollment. Pregnancy information such as morbidity symptoms were collected monthly throughout pregnancy, including morbidity recall and measurement of pulse, temperature and blood pressure. As soon as possible after birth, study staff interviewed the mother about labor and delivery and recorded the pregnancy outcome (e.g., live births, stillbirths, multiple births). Awareness of a government cash transfer program that incentivized women to receive ANC and deliver at a facility were noted through maternal recall.

Women’s and their husbands’ occupations were grouped into four categories: (1) farmer, (2) does not work outside home, (3) unskilled or day or contracted laborer, and (4) business or private or government service. Maternal education was divided into three levels: none, 1–5 years, and over 5 years of formal education. Wealth was classified into 5 quintiles based on assets and living conditions (8, 15). Caste/religion was categorized into 4 groups: Brahmin and Chhetri, Vaishya, Shudra, and Muslim and others, with Brahmin and Chhetri being the two highest castes in the study setting. Maternal age groups were: <18 years, 18–35, and > 35 years of age at enrollment. Maternal height was divided into 3 ranges: < 145 centimeters (cm),, 145–150 cm, and ≥ 150 cm. Gestational age was classified as moderately preterm (< 34 weeks), mildly preterm (34–37 weeks), and term (≥ 37 weeks). Parity was categorized into 3 groups: no prior pregnancy, prior pregnancy but no live or stillbirth, and parity ≥ 1. Pregnancy history/outcome along with survival of any babies born was divided into 4 groups: prior live birth (LB) but not died, prior LB died, prior pregnancy but no LB, and no prior pregnancy. Prior pregnancy history concerning stillbirth (SB) was divided into 3 categories: prior pregnancy but no SB, prior SB, and no prior pregnancy. Interpregnancy interval (IPI) was divided into 4 groups: 18 to 36 months, < 18 months, > 36 months, and no prior pregnancy. Maternal morbidities like fever, tachycardia, hypertension, respiratory illnesses, swelling of hands and face, gastrointestinal symptoms, vaginal bleeding, and sexually transmitted illness (STI) were binary variables (present at any time during second or third trimesters). Detailed explanations for predictors are shown in appendix Table 1.

### Statistical analyses

To examine factors associated with the completion of ≥ 4 ANC visits, we fit Generalized Estimating Equation (GEE) models with a logit link and an exchangeable working correlation structure, to account for the within-person correlation arising from including more than one pregnancy for many women in the study. We presented both the crude and adjusted odds ratio (OR) of ≥ 4 ANC visits with the corresponding 95% confidence intervals (CIs). All analyses were conducted using Stata software version SP 17.0 (StataCorp. 2021).

## Results

The distributions of population characteristics are shown in Table 1, overall, and by number of ANC visits < 4 and ≥ 4. A total of 31,867 pregnancies with live birth outcomes from 24,428 women were included in our analyses. A tenth (11.0%, n = 3,517) of pregnancies were missing the reported number of ANC visits. In our study population, accounting for pregnancies with missing number of ANC visits, there were 28.0% with ≥ 4 ANC visits and 61.0% pregnancies with < 4 ANC visits, with the average number of ANC visits in the whole analyses sample being 2.4 ± 1.8. Among 28,350 pregnancies with non-missing numbers of ANC visits, there were 31.4% with ≥ 4 ANC visits and 68.6% pregnancies with < 4 ANC visits. Specifically, out of 19,443 pregnancies with fewer than four ANC visits, 28.4% had no ANC visits, 21.3% had one ANC visit, 26.1% had two ANC visits, and 24.2% had three ANC visits.

Results of the GEE regression model (i.e. crude and adjusted OR, 95% CI) are summarized in Table 2 and are shown below.

### Results: Socio-economic and demographic factors

Women engaged in non-agricultural professions, such as business, private service, or government service, were more likely to have ≥ 4 ANC visits than women who were farmers (adjusted OR = 1.52, 95% CI [1.19, 1.93]). Women whose husbands worked in business, private, or government service were more likely to attend ≥ 4 ANC visits compared to those whose husbands were farmers (adjusted OR = 1.28, 95% CI [1.19, 1.39]). Moreover, women with husbands working as unskilled laborers, day laborers, or contracted laborers were more likely to have ≥ 4 ANC visits when compared to the farmer husband group (adjusted OR = 1.10, 95% CI [1.02, 1.18]).

Women between 18 and 35 years of age had the highest probability of attending ≥ 4 ANC sessions. In comparison to this age group, women younger than 18 years or those older than 35 years were less likely to attend 4 + ANC visits (adjusted OR = 0.81, 95% CI [0.74, 0.88]) and (adjusted OR = 0.77, 95% CI [0.62, 0.96]), respectively. Taller women had a higher probability of attending ≥ 4 ANC sessions (adjusted OR = 1.15, 95% CI [1.05, 1.26]) for those between 145 cm and 150 cm, and those ≥ 150 cm (adjusted OR = 1.17, 95% CI [1.07, 1.27]) when compared to women shorter than 145 cm. Compared to the upper castes of Brahmin and Chhetri, Vaishya, Shudra, and Muslim and others women had 34%, 44%, and 33% lower odds of attending ≥ 4 ANC sessions.

Women with one to five years of education were more likely to have ≥ 4 ANC sessions than those without any education (adjusted OR = 1.38, 95% CI [1.26, 1.53])). Those with over five years of education were more likely to attend ≥ 4 ANC visits compared to their non-educated counterparts (adjusted OR = 1.79, 95% CI [1.66, 1.93]). While there was no consistent increasing trend across wealth quintiles, women from the wealthiest quintile were more likely to attend ≥ 4 ANC visits compared to those from the poorest quintile (adjusted OR = 1.40, 95% CI [1.26, 1.55).

From 2010 to 2017, women had an increased probability of attending ≥ 4 ANC visits per year (adjusted OR = 1.19, 95% CI [1.17, 1.22]). The prevalence of ≥ 4 ANC visits by child’s birth year increased across time as follows: 64 (16.9%) in 2010, 510 (18.1%) in 2011, 714 (23.7%) in 2012, 576 (19.9%) in 2013, 1,836 (31.6%) in 2014, 2,328 (40.8%) in 2015, 2,689 (37.0%) in 2016, and 190 (43.1%) in 2017, not including missing values in the denominator.

Additionally, women who were aware of the government’s conditional cash transfer program promoting ≥ 4 ANC visits and facility-based delivery were more likely to attend ≥ 4 ANC visits compared to those who were unaware of the program (adjusted OR = 2.26, 95% CI [2.01, 2.54]). The prevalence of awareness of the government’s conditional cash transfer program promoting ≥ 4 ANC visits increased over time: 254 (66.8%) in 2010, 2,286 (82.1%) in 2011, 2,472 (84.3%) in 2012, 2,470 (88.1%) in 2013, 5,408 (93.7%) in 2014, 5,400 (94.7%) in 2015, 6,891 (95.1%) in 2016, and 428 (96.8%) in 2017, not including missing values in the denominator.

### Results: Reproductive history related factors

Women with mildly preterm (34–37 weeks) and moderately preterm (< 34 weeks) births were less likely to have attended ≥ 4 ANC visits compared to term births (adjusted OR = 0.67, 95% CI [0.61, 0.73]) for mildly preterm; and for moderately preterm (adjusted OR = 0.41, 95% CI [0.35, 0.49])). Women with ≥ 1 parity were less likely to attend ≥ 4 ANC visits than those with no prior pregnancies (adjusted OR = 0.66, 95% CI [0.61, 0.72]). Similarly, those with a previous pregnancy but no prior live birth were more likely to attend ≥ 4 ANC visits than those with a previous live birth (adjusted OR = 2.14, 95% CI [1.57, 2.92]). Women with IPI of > 36 months were more likely to attend ≥ 4 ANC visits compared to those with an IPI of 18–36 months (OR = 1.31, 95% CI [1.19, 1.45]).

### Results: Morbidity during pregnancy

Women who experienced hypertension at least once during their second or third trimesters were less likely to have attended ≥ 4 ANC visits compared to those who did not experience hypertension (adjusted OR = 0.79, 95% CI [0.69, 0.90]). Pregnancy-related morbidities, including respiratory problems, poor appetite, vaginal bleeding, swelling of hands and face, and STIs, were associated with statistically significant increased odds of ≥ 4 ANC visits and respective OR are listed in Table 2.

## Discussion

### Socioeconomic and demographic factors

We observed multiple interrelated factors associated with the receipt of ≥ 4 ANC visits. These factors included SES, demographic characteristics, women’s awareness of the conditional cash transfer program, pregnancy history, and pregnancy-related morbidities. We found that women engaged in business, private, or government services had a higher likelihood of receiving ≥ 4 ANC visits, as did women with husbands in non-agricultural sectors. This suggests that household members’ occupations, indicative of their financial situation, influence women’s access and utilization of healthcare services during pregnancy in Nepal.

We found a positive dose response between higher educational levels and the receipt of ≥ 4 ANC visits. Prior studies using NDHS 2001 and 2011 data align with our findings, emphasizing the role of education in facilitating the use of maternal health services (8, 13). Higher education enhances women’s understanding of the importance of professional healthcare, broadening their accessibility and utilization (10, 16). In our study, women from wealthier households were more likely to have attended ≥ 4 ANC visits, which corresponded to results from previous studies (10).

Religion and caste were associated with the receipt of ≥ 4 ANC visits. In Nepal, Brahmin and Chhetri are considered to be of higher caste than Vaishya and Shudra (17). Our findings suggest that Brahmin and Chhetri women were more likely to attend ≥ 4 ANC visits compared to those identified as Vaishya, Shudra, or Muslim.

Maternal age influenced ANC visits. Women under 18 or over 35 years of age were less likely to have ≥ 4 visits, independent of parity, although younger women were more likely to be parity zero and older women multiparous. Taller women were more inclined to have had at least four ANC visits, compared to women shorter than 145 cm. Prior studies suggest that shorter women had greater risk of preterm, delivering low birth weight babies, and neonatal mortality, which indicated maternal height as a direct determinant of adverse birth outcomes (5, 8, 18, 19). Our results suggest that maternal height and SES were independently associated with ANC attendance, even though these two variables may be correlated.

Awareness of the government conditional cash transfer program influenced the likelihood of attending ≥ 4 ANC visits. The Aama Surakshya Program (conditional cash transfer program) was a cash-based incentive program introduced by the Government of Nepal (GoN) to increase ANC attendance, promote institutional deliveries, and reduce maternal morbidity and mortality (20). The GoN reported that the Aama program had a positive impact on institutional deliveries for pregnant women in Nepal in 2020, with a strong relationship between the program and completing ≥ 4 ANC visits (20). Our result reinforced the GoN report results. Furthermore, our results suggested that the odds of ≥ 4 ANC visits increased from 2010 through 2017, which likely reflects that more women knew about the conditional cash transfer program and made more use of it over this time period.

### Pregnancy history and prior pregnancy outcomes

Our study revealed that first-time pregnant women were more likely to have ≥ 4 ANC visits compared to those with parity ≥ 1 or previous pregnancy but no live or stillbirths. This finding is similar to one from a population-based study using 2011 Nepal DHS data which found that the odds of receiving good quality ANC decreased by 21% for every additional pregnancy that resulted in a live or stillbirth (10). The number of ANC visits might be associated with the continuity of ANC, which was a component of the quality of ANC. We observed that women with prior stillbirth histories might be more likely to attend ≥ 4 ANC visits, albeit not statistically significant, which may be due to the low prevalence of prior stillbirths (4.3%) in our study. Our findings suggest that fear or concern due to past experiences of adverse birth outcomes may increase the receipt of ≥ 4 ANC visits.

We identified gestational age at delivery as a factor associated with attending ≥ 4 ANC visits. In Nepal, the suggested schedule for the four ANC visits is distributed across the 4th (12–16 weeks), 6th (20–24 weeks), 8th (28–32 weeks), and 9th (36–40 weeks) months of pregnancy (21). Consequently, a pregnancy culminating at 33 weeks or less would reduce the likelihood of attending four or more ANC visits, especially given that the fourth ANC visit is typically scheduled for the 9th month. Prior studies indicate that high quality ANC is important in mitigating preterm labor (10–12). Thus, efforts in prompting women to attend more ANC, coupled with enhancements in quality of ANC care, may reduce preterm birth. We did not measure the quality of the ANC in this study, only receipt of care.

We also found that women with interpregnancy intervals exceeding 36 months were more likely to have attended ≥ 4 ANC visits. One reason may be that women with longer IPIs may perceive their current pregnancy to be higher risk. Alternatively, longer IPIs may be associated with suboptimal health of these women, prompting them to seek increased ANC. A study conducted in Nepal found that both very short IPIs (less than 18 months) and longer IPIs (beyond 59 months) were associated with higher risks of poor maternal and perinatal outcomes (22).

In our study, women who had multiple birth were more likely to attend ≥ 4 ANC visits. However, given the few multiple gestational pregnancies in our sample (n = 256, representing 0.8%), this association was not statistically significant. One possible explanation is that if women were informed about their multiple gestation, they might be more proactive in seeking ANC. Although rare in this study, ultrasound is becoming more common in this area, and relatively early knowledge of multiple gestation may be increasing (23).

### Morbidity symptoms in pregnancy

Our study indicates that during the 2nd or 3rd trimesters, women who reported any of the following symptoms: respiratory issues, swelling of the hands and face, vaginal bleeding, symptoms like poor appetite, nausea, vomiting, or an STI, were more likely to attend ≥ 4 ANC visits. Women with signs and symptoms of morbidity would be more likely to seek care in pregnancy more frequently than those who feel healthy. In addition, the NOMS study protocol directed study workers to refer women reporting certain gestational morbidity symptoms to health facilities, though compliance with this referral was not tracked in our data. Conversely, women who had at least one hypertensive measurement were less likely to attend ≥ 4 ANC visits. Even though women were told by the study team if they had hypertensive measurements, given that this is often without noticeable symptoms, affected women might not perceive an immediate need to seek further care.

### Strengths and limitations

One of the strengths of our study was the utilization of a population-based pregnancy cohort dataset, enabling a comprehensive examination of potential risk factors measured prospectively throughout pregnancy However, due to the limitations with observational studies, establishing a causal relationship between the factors discussed and the attendance of four or more ANC visits remains challenging. Also, we may have confounding such as unmeasured medical conditions during follow-up visits. We also asked the question about number of ANC visits with the exact wording used in the Nepal DHS. However, another study in this setting found that the validity of maternal recall of the number of ANC visits at six months postpartum is poor, although we do not know its validity when being asked at the time of delivery as in our study (24). In addition, the external generalizability may be low for this study given that factors contributing to the receipt of ≥ 4 ANC visits, particularly in urban areas, might be different. Recall bias is a potential limitation, as certain factors were ascertained through retrospective questioning. Most of our information is based on self-reported answers, introducing the possibility of response biases, including social desirability bias. Only the number of ANC visits was collected prospectively, and there was no evaluation of ANC quality. The presence of missing data for certain characteristics could introduce bias. Specifically, we did not have much morbidity data during the first trimester because it was difficult to identify and enroll women so early in pregnancy. Hence first trimester morbidity was excluded from the morbidity analysis.

## Conclusions

In summary, multiple SES factors are strongly associated with higher probability of the receipt of ≥ 4 ANC visits in Nepal. In addition, programs such as the Aama Surakshya have increased ANC attendance, independent of SES factors. However, the positive association of knowledge of Aama Surakshya and ≥ 4 ANC visit may be confounded by other unmeasured factors, which is always a concern with observational studies.

Occupations of women and their husbands, maternal age and height, caste/religion, maternal education, wealth quintile, preterm birth, no prior pregnancy or prior pregnancy but 0 parity, longer interpregnancy intervals, deaths of prior live births, women’s knowledge about the conditional cash transfer program, and gestational morbidity symptoms were significantly associated with attendance at ≥ 4 ANC visits.

We did not examine the quality of ANC nor whether attending 4 or more ANC visits led to improved outcomes. However, assuming that more ANC visits are beneficial, promotion of the conditional cash transfer program would likely increase ANC attendance. Educating pregnant women about morbidity signs and symptoms during pregnancy and promotion of ANC attendance may increase ANC service utilization, and possibly reduce maternal and neonatal mortality.

Our identification of factors associated with attendance of ≥ 4 ANC visits in Nepal may help design programs to increase ANC uptake. The 2022 NDHS reports that 81% women received ≥ 4 ANC visits and 94% women had at least one ANC from a skilled provider (4). Given that only 28% of pregnancies in our study reported ≥ 4 ANC visits, there is significant potential for improvement in this coverage. In Nepal, the adult literacy rate for women is 60%, lower than the 68% recorded for men (25). Promoting education, particularly for girls, might increase ANC utilization. Health education campaigns targeted at illiterate women with financial incentives, may be short-term interventions that improve ANC attendances (26). Longer term interventions such as advocating for women’s education, employment, and broader empowerment may increase ANC attendance and overall maternal and neonatal health in Nepal (22).

Research has shown that higher education of husbands, with their increased involvement during their wives’ pregnancies, can increase the likelihood of women attending ≥ 4 ANC visits, thereby benefiting maternal health (10). Despite these findings, there is a limited body of research exploring the connection between the education level of husbands and ANC visits. Our study revealed that women, whose husbands were employed in non-agricultural sectors, were more likely to attend ≥ 4 ANC visits. This indicates the role husbands play in maternal health, independent of the wife’s educational background. We were not able to examine the role of other family members such as the mother-in-law in influencing ANC visit uptake. In addition to these factors that could increase ANC utilization, quality of ANC would also be important to examine, as women may be more likely to seek ANC services if they perceive a health benefit. At the time of this study, Nepal’s policy was to promote 4 ANC visits in pregnancy, but WHO is now recommending 8 or more contacts during pregnancy (21, 27). With changing recommendations, it would be important to examine factors that are associated with ANC attendance at the new recommendation of 8 visits.

## Figures and Tables

**Table 1:** Characteristics of the whole analyses sample and stratified by ≥ 4 ANC visits or not

	Total	0–3 ANC	≥ 4 ANC
	N = 28,350	N = 19,443	N = 8,907
**Women occupation**			
Farmer	1,558	1,162 (74.58%)	396 (25.42%)
Does not work outside home	25,639	17,492 (68.22%)	8,147 (31.78%)
Unskilled laborer/day labor or contracted laborer	730	588 (80.55%)	142 (19.45%)
Business or private or government service	395	181 (45.82%)	214 (54.18%)
Missing	28	20 (71.43%)	8 (28.57%)
**Husband occupation**			
Farmer	7,745	5,465 (70.56%)	2,280 (29.44%)
Does not work outside home	729	450 (61.73%)	279 (38.27%)
Unskilled laborer/day labor or contracted laborer	13,261	9,550 (72.02%)	3,711 (27.98%)
Business or private or government service	6,580	3,957 (60.14%)	2,623 (39.86%)
Missing	35	21 (60.00%)	14 (40.00%)
**Maternal age**			
18 to 35	23,365	15,954 (68.28%)	7,411 (31.72%)
Less than 18	4,315	2,966 (68.74%)	1,349 (31.26%)
More than 35	668	521 (77.99%)	147 (22.01%)
Missing	2	2 (100.00%)	(0.00%)
**Maternal Height (centimeter)**			
< 145	4,186	3,096 (73.96%)	1,090 (26.04%)
≥ 145 – <150	8,509	5,915 (69.51%)	2,594 (30.49%)
≥ 150	15,609	10,397 (66.61%)	5,212 (33.39%)
Missing	46	35 (76.09%)	11 (23.91%)
**Caste/Religion**			
Brahmin and Chhetri	863	449 (52.03%)	414 (47.97%)
Vaishya	20,429	13,809 (67.60%)	6,620 (32.40%)
Shudra	4,431	3,368 (76.01%)	1,063 (23.99%)
Muslim and other religions	2,599	1,795 (69.07%)	804 (30.93%)
Missing	28	22 (78.57%)	6 (21.43%)
**Mother’s Education**			
No schooling	19,360	14,434 (74.56%)	4,926 (25.44%)
1 to 5 years	2,392	1,520 (63.55%)	872 (36.45%)
More than 5 years	6,569	3,468 (52.79%)	3,101 (47.21%)
Missing	29	21 (72.41%)	8 (27.59%)
**Wealth quintile**			
Poorest	5,875	4,660 (79.32%)	1,215 (20.68%)
Poorer	5,753	4,171 (72.50%)	1,582 (27.50%)
Middle	5,640	3,825 (67.82%)	1,815 (32.18%)
Richer	5,545	3,504 (63.19%)	2,041 (36.81%)
Richest	5,518	3,269 (59.24%)	2,249 (40.76%)
Missing	19	14 (73.68%)	5 (26.32%)
**Gestational age (weeks)**			
Term: ≥ 37 weeks	24,119	16,099 (66.75%)	8,020 (33.25%)
Mildly preterm: 34–37 weeks	3,059	2,347 (76.72%)	712 (23.28%)
Moderately preterm: < 34 weeks	1,169	994 (85.03%)	175 (14.97%)
Missing	3	3 (100.00%)	(0.00%)
**Multiple births**			
Singleton	28,120	19,274 (68.54%)	8,846 (31.46%)
Twin/Triplet	230	169 (73.48%)	61 (26.52%)
**Parity**			
No Prior Pregnant	7,917	4,907 (61.98%)	3,010 (38.02%)
Prior pregnancy but no live or stillbirth	687	361 (52.55%)	326 (47.45%)
Parity ≥ 1	19,591	14,062 (71.78%)	5,529 (28.22%)
Missing	155	113 (72.90%)	42 (27.10%)
**Interpregnancy interval**			
18 to 36 months	7,204	5,211 (72.33%)	1,993 (27.67%)
Less than 18 months	10,274	7,402 (72.05%)	2,872 (27.95%)
More than 36 months	2,942	1,911 (64.96%)	1,031 (35.04%)
No Prior Pregnancy	7,917	4,907 (61.98%)	3,010 (38.02%)
Missing	13	12 (92.31%)	1 (7.69%)
**Any Prior live birth (LB) died**			
Prior LB but not died	15,772	11,276 (71.49%)	4,496 (28.51%)
Prior LB died	3,291	2,442 (74.20%)	849 (25.80%)
Prior Pregnancy but no LB	929	490 (52.74%)	439 (47.26%)
No prior pregnancy	7,917	4,907 (61.98%)	3,010 (38.02%)
Missing	441	328 (74.38%)	113 (25.62%)
**Any prior pregnancy ended in stillbirth (SB)**			
Prior pregnancy but no SB	19,178	13,681 (71.34%)	5,497 (28.66%)
Prior SB	1,239	846 (68.28%)	393 (31.72%)
No prior pregnancy	7,917	4,907 (61.98%)	3,010 (38.02%)
Missing	16	9 (56.25%)	7 (43.75%)
**Gestational weight gains**	4	3 (96.06%)	4 (108.17%)
**Knowledge of conditional cash transfer program**			
No	2,450	2,131 (86.98%)	319 (13.02%)
Yes	25,565	17,013 (66.55%)	8,552 (33.45%)
Missing	335	299 (89.25%)	36 (10.75%)
**Year of childbirth**			
2010	378	314 (83.07%)	64 (16.93%)
2011	2,821	2,311 (81.92%)	510 (18.08%)
2012	3,013	2,299 (76.30%)	714 (23.70%)
2013	2,892	2,316 (80.08%)	576 (19.92%)
2014	5,820	3,984 (68.45%)	1,836 (31.55%)
2015	5,713	3,385 (59.25%)	2,328 (40.75%)
2016	7,270	4,581 (63.01%)	2,689 (36.99%)
2017	441	251 (56.92%)	190 (43.08%)
Missing	2	2 (100.00%)	(0.00%)
**Fever**			
No	25,256	17,288 (68.45%)	7,968 (31.55%)
Yes	2,987	2,075 (69.47%)	912 (30.53%)
Missing	107	80 (74.77%)	27 (25.23%)
**Tachycardia**			
No	12,508	8,584 (68.63%)	3,924 (31.37%)
Yes	15,757	10,795 (68.51%)	4,962 (31.49%)
Missing	85	64 (75.29%)	21 (24.71%)
**Hypertension**			
No	26,996	18,447 (68.33%)	8,549 (31.67%)
Yes	1,274	936 (73.47%)	338 (26.53%)
Missing	80	60 (75.00%)	20 (25.00%)
**Poor appetite, nausea & vomiting**			
No	14,559	10,397 (71.41%)	4,162 (28.59%)
Yes	13,709	8,984 (65.53%)	4,725 (34.47%)
Missing	82	62 (75.61%)	20 (24.39%)
**Respiratory problem**			(0.00%)
No	17,853	12,486 (69.94%)	5,367 (30.06%)
Yes	10,415	6,895 (66.20%)	3,520 (33.80%)
Missing	82	62 (75.61%)	20 (24.39%)
**Swelling (hands or face)**			
No	26,790	18,440 (68.83%)	8,350 (31.17%)
Yes	1,479	942 (63.69%)	537 (36.31%)
Missing	81	61 (75.31%)	20 (24.69%)
**Sexually transmitted illness**			
No	22,293	15,476 (69.42%)	6,817 (30.58%)
Yes	5,975	3,905 (65.36%)	2,070 (34.64%)
Missing	82	62 (75.61%)	20 (24.39%)
**Vaginal bleeding**			
No	27,775	19,080 (68.69%)	8,695 (31.31%)
Yes	493	301 (61.05%)	192 (38.95%)
Missing	82	62 (75.61%)	20 (24.39%)

Note: LB: livebirth; SB: stillbirth

* Morbidity symptoms in pregnancy are for presence at any time in either the 2nd and/or 3rd trimesters

**Table 2:** Longitudinal risk factors for ≥ 4 ANC visits

	Crude Odds Ratio	95% CI	Adjusted Odds Ratio	95% CI
***Women occupation***				
Farmer	Reference	Reference	Reference	Reference
Does not work	**1.31** **	**(1.17,1.46)**	0.91	(0.81, 1.04)
Unskilled/day labor/contracted laborer	**0.76** **	**(0.62, 0.92)**	0.94	(0.75, 1.18)
Business/private/government service	**3.16** **	**(2.52, 3.95)**	**1.52** **	(1.19, 1.93)
**Husband occupation**				
Farmer	Reference	Reference	Reference	Reference
Does not work	**1.43** **	**(1.22, 1.66)**	1.14	(0.96, 1.35)
Unskilled laborer/day labor or contracted laborer	**0.94** *	**(0.88, 1.00)**	**1.10** *	**(102, 1.18)**
Business/private service/government service	**1.54** **	**(144, 1.65)**	**1.28** **	**(119, 1.39)**
**Maternal age (years)**				
18–35	Reference	Reference	Reference	Reference
< 18	0.96	(0.90, 1.03)	**0.81** **	**(0.74, 0.88)**
> 35	**0.61** **	**(0.50, 0.73)**	**0.77** *	**(0.62, 0.96)**
**Maternal height (cm)**				
< 145	Reference	Reference	Reference	Reference
145–150	**1.25** **	**(1.15, 1.36)**	**1.15** **	**(1.05, 1.26)**
≥ 150	**1.42** **	**(131, 1.54)**	**1.17** **	**(1.07, 1.27)**
**Caste/ethnicity**				
Brahmin and Chhetri	Reference	Reference	Reference	Reference
Vaishya	**0.52** **	**(0.46, 0.60)**	**0.66** **	**(0.57, 0.77)**
Shudra	**0.35** **	**(0.30, 0.40)**	**0.56** **	**(0.47, 0.67)**
Muslim and others	**0.48** **	**(0.41, 0.56)**	**0.67** **	**(0.56, 0.80)**
**Women education (years)**				
No schooling	Reference	Reference	Reference	Reference
1–5	**1.64** **	**(1.50, 1.80)**	**1.38** **	**(1.26, 1.53)**
> 5	**2.61** **	**(2–46, 2.78)**	**1.79** **	**(1.66, 1.93)**
**Wealth quintile**				
Poorest	Reference	Reference	Reference	Reference
Poorer	**1.41** **	**(1.30, 1.53)**	**1.19** **	**(1.09, 1.31)**
Middle	**1.77** **	**(1.63, 1.92)**	**1.39** **	**(1.26, 1.52)**
Richer	**2.14** **	**(1.97, 2.33)**	**1.44** **	**(1.31, 1.59)**
Richest	**2.56** **	**(2.35, 2.78)**	**1.40** **	**(1.26, 1.55)**
**Gestational age (weeks)**				
Term (≥ 37)	Reference	Reference	Reference	Reference
Mildly preterm (34–37)	**0.63** **	**(0.58, 0.68)**	**0.67** **	**(0.61, 0.73)**
Moderately preterm (< 34)	**0.38** **	**(0.32, 0.44)**	**0.41** **	**(0.35, 0.49)**
**Multiple birth**				
Singleton	Reference	Reference	Reference	Reference
Twin/Triplet	0.80	(0.60, 1.07)	1.20	(0.88, 1.64)
Parity				
No Prior Pregnancy	Reference	Reference	Reference	Reference
Prior pregnancy but no live or stillbirth	**1.47** **	**(1.26, 1.72)**	0.73	(0.52, 1.04)
Parity ≥ 1	**0.68** **	**(0.65, 0.72)**	**0.66** **	**(0.61, 0.72)**
**Interpregnancy Interval (months)**				
18 to 36 months	Reference	Reference	Reference	Reference
< 18	1.01	(0.94, 1.07)	0.96	(0.89, 1.03)
> 36	**1.37** **	**(1.25, 1.49)**	**1.31** **	**(1.19, 1.45)**
No prior pregnancy	**1.49** **	**(1.40, 1.60)**	-	-
**Prior live birth (LB) died**				
Prior LB but not died	Reference	Reference	Reference	Reference
Prior LB died	**0.90** *	**(0.82, 0.97)**	1.05	(0.96, 1.15)
Prior pregnancy but no LB	**2.12** **	**(1.86, 2.42)**	**2.14** **	**(15.7, 2.92)**
No prior pregnancy	**1.45** **	**(1.37, 1.53)**	-	-
**Prior pregnancy ended in stillbirth (SB)**				
Prior pregnancy but no SB	Reference	Reference	Reference	Reference
Prior SB	**1.14** *	**(1.01, 1.29)**	1.12	(0.95, 1.32)
No prior pregnancy	**1.43** **	**(1.36, 1.51)**	-	-
**Conditional cash transfer program knowledge**	**2.96** **	**(2.66, 3.30)**	**2.26** **	**(2.01, 2.54)**
**Year of childbirth**	**1.22** **	**(1.20, 1.23)**	**1.19** **	**(1.17, 1.22)**
**Fever**				
No	Reference	Reference	Reference	Reference
Yes	0.98	(0.90, 1.06)	1.05	(0.96, 1.15)
**Tachycardia**				
No	Reference	Reference	Reference	Reference
Yes	1.01	(0.96, 1.06)	0.99	(0.93, 1.04)
**Hypertension**				
No	Reference	Reference	Reference	Reference
Yes	**0.78** **	**(0.69, 0.88)**	**0.79** **	**(0.69, 0.90)**
**Respiratory problem**				
No	Reference	Reference	Reference	Reference
Yes	**1.18** **	**(1.12, 1.24)**	**1.12** **	**(1.06, 1.19)**
**Poor appetite, nausea & vomiting**				
No	Reference	Reference	Reference	Reference
Yes	**1.29** **	**(1.23, 1.36)**	**1.17** **	**(1.11, 1.24)**
**Vaginal bleeding**				
No	Reference	Reference	Reference	Reference
Yes	**1.34** **	**(1.12, 1.61)**	**1.35** **	**(1.11, 1.65)**
**Swelling of hands and face**				
No	Reference	Reference	Reference	Reference
Yes	**1.25** **	**(1.13, 1.40)**	**1.26** **	**(1.12, 1.41)**
**Sexually transmitted illness**				
No	Reference	Reference	Reference	Reference
Yes	**1.19** **	**(1.12, 1.27)**	**1.24** **	**(1.16, 1.33)**

Note:

** p< 0.01,

* p < 0.05

* Morbidity symptoms in pregnancy are for presence at any time in either the 2nd and/or 3rd trimesters

## Data Availability

Data are available through Synapse Open Source Collaboration Platform at https://www.kiglobalhealth.org/

## Appendix Table 1. Explanations of Covariates

**Table:**

Covariates	Explanations
**Women/Husband occupation**	Occupations of women and their husbands were each categorized into four groups: “Farmer” (reference), “Does not work outside home”, “Unskilled laborer/day labor or contracted laborer”, and “Business or private or government service”.
**Maternal age (years)**	Maternal age groups were: 18 to 35 (reference), below 18, and over 35.
**Maternal height (centimeters)**	Maternal height was categorized into 3 groups: < 145 cm (reference), 145–150 cm, and ≥150 cm.
**Caste/Religion**	Caste/religion was categorized into 4 groups: Brahmin and Chhetri (reference), Vaishya, Shudra, and Muslim and others, with Brahmin and Chhetri being the two highest castes for those of the Hindu religion.
**Mother’s education (years)**	Maternal education was divided into 3 groups: No schooling (reference), 1 to 5 years, and more than 5 years.
**Wealth quintile**	Wealth was classified into 5 quintiles, ranging from poorest, poorer, middle, richer to richest. The wealth index was constructed from ownership of land and household goods, house construction, latrine type and water source, and whether the home had electricity.
**Gestational age (weeks)**	Gestational age was categorized into 3 groups: “Moderately preterm: < 34 weeks”, “Mildly preterm: ≥ 34–37 weeks” and “Term: ≥ 37 weeks”.
**Multiple birth**	Multiple birth was a binary variable: singleton (reference), and twin or triplet.
**Parity**	Parity denoted the number of times that a woman had given birth to a baby of at least 28 weeks of gestational age, regardless of whether it was a live birth or stillbirth. The parity variable “parity including both live birth and stillbirth at enrollment” was categorized into 3 groups: “No Prior Pregnancy” (reference), “ Prior pregnancy but no live or stillbirth”, and “Parity ≥ 1”.
**Interpregnancy interval (months)**	Interpregnancy Interval was categorized into 4 groups: 18 to 36 months (reference), less than 18 months, more than 36 months, and no prior pregnancy.
**Any Prior live birth (LB) died**	Any prior LB died was categorized as 4 groups: “Prior LB but not died” (reference), “Prior LB died”, “Prior Pregnancy but no LB”, and “No prior pregnancy”.
**Any prior pregnancy ended in stillbirth (Sb)**	Any prior pregnancy ended in SB was categorized into 3 groups: “Prior pregnancy but no SB” (reference), “Prior SB”, and “No prior pregnancy”.
**Knowledge of conditional cash transfer program**	Awareness about the “Conditional cash transfer program” was a binary response, indicating whether the woman was familiar with the government’s conditional cash transfer program that pays women if they have 4 or more ANC visits and an additional payment for delivery at a facility.
**Year of childbirth**	“Year of birth of the child” indicated the exact year of the birth of the child during each pregnancy, ranging from 2010 to 2017.
**Morbidity indicators**	
**Fever**	Fever was defined as a temperature of at least 99 Fahrenheit during the second or third trimesters, recorded during monthly visits.
**Tachycardia**	Tachycardia referred to a pulse rate of 100 beats per minute or higher measured at monthly visits during these trimesters.
**Hypertension**	Hypertension was identified by a systolic blood pressure above 140 mmHg or a diastolic pressure above 90 mmHg in the latter two trimesters.
**Poor appetite, nausea & vomiting**	Upper gastrointestinal (GI) symptoms included poor appetite, vomiting, and nausea, while lower GI symptoms involved watery stools or blood/mucus presence.
**Respiratory problem**	Respiratory illness included symptoms like coughing, breathlessness, wheezing, or shortness of breath.
**Swelling of hands or face**	Swelling symptoms referred only to swelling of hands and face to indicate possible pre-eclampsia; leg and foot swelling was omitted due to it being common in pregnancy.
**Vaginal bleeding**	Vaginal bleeding meant any bleeding during pregnancy.
**Sexually transmitted illness (STI)**	STI was identified by painful urination or a foul-smelling vaginal discharge.

Note: All mentioned morbidity indicators were binary and based on maternal recall in the past 30 days, except for blood pressure, pulse and temperature, which were measured at each monthly visit during pregnancy.
